# Onset of a Declining Trend in Fatal Motor Vehicle Crashes Involving Drunk-driving in Japan

**DOI:** 10.2188/jea.JE20120134

**Published:** 2013-05-05

**Authors:** Shinji Nakahara, Kota Katanoda, Masao Ichikawa

**Affiliations:** 1 Department of Epidemiology and Health Promotion, St. Mariana University, Kawasaki, Kanagawa, Japan; 1 聖マリアンナ医科大学; 2 Center for Cancer Control and Information Services, National Cancer Center, Tokyo, Japan; 2 国立がん研究センター がん対策研究センター; 3 Faculty of Medicine, University of Tsukuba, Tsukuba, Ibaraki, Japan; 3 筑波大学 医学医療系

**Keywords:** alcohol, Japan, mass media, regression analysis, traffic accidents

## Abstract

**Background:**

In Japan, introduction of severe drunk-driving penalties and a lower blood alcohol concentration (BAC) limit in June 2002 was followed by a substantial reduction in fatal alcohol-related crashes. However, previous research suggests that this reduction started before the legal amendments. The causes of the decrease have not been studied in detail.

**Methods:**

Monthly police data on fatal road traffic crashes from January 1995 to August 2006 were analyzed using a joinpoint regression model to identify change-points in the trends of the proportion of drunk-driving among drivers primarily responsible for fatal crashes. We analyzed the data by BAC level (≥0.5 or <0.5 mg/ml), then conducted analyses stratified by vehicle type (car or motorcycle) and age group (<45 or ≥45 years) only for the proportion of those with a BAC of 0.5 mg/ml or higher.

**Results:**

Among all drivers, the proportion of those with a BAC of 0.5 mg/ml or higher and those with a BAC greater than 0 but less than 0.5 mg/ml showed a change-point from increase to decrease in February 2000 and in May 2002, respectively. The proportion of those with a BAC of 0.5 mg/ml or higher showed a change-point from increase to decrease in October 1999 among car drivers and in April 2000 among drivers younger than 45 years. There was no change-point among motorcyclists. A change-point from no trend to a decrease in January 2002 was observed among those 45 years or older.

**Conclusions:**

The change-point identified around the end of 1999 to the start of 2000 suggests that a high-profile fatal crash in November 1999, which drew media attention and provoked public debate, triggered subsequent changes in drunk-driving behavior.

## Abstract (ja)

**【背景】:**

日本におけるアルコールが関与する死亡交通事故は、2002年6月の道路交通法改正による飲酒運転に対する罰則の強化と、罰則適用基準値の引き下げにより、大きく減少した。しかし、先行研究ではこの減少傾向が法改正よりも前に始まった可能性が指摘されている。この減少傾向の原因については十分な分析が行われていない。

**【方法】:**

分析には1995年1月から2006年8月までの、警察庁集計による月別死亡交通事故データを用いた。死亡事故の第一当事者（当該事故で最も責任の重いと考えられた運転者）が飲酒をしていた割合を従属変数とし、そのトレンドの変曲点をジョインポイント回帰分析により求めた。分析はまず、全第一当事者に占める血中アルコール濃度（Blood alcohol concentration: BAC）が0.5mg/ml以上と0.5mg/ml未満（道路交通法は血中濃度にかかわらず酒気を帯びての運転を禁じており、罰則適用基準値未満でも飲酒運転として事故データに記録される）の割合について行った。次に、車種別（四輪車、二輪車）、年齢別（45歳以上、45歳未満）に層別化し、0.5mg/ml以上の割合について分析した。

**【結果】:**

全第一当事者に占める、BACが0.5mg/ml以上の飲酒運転の割合のトレンドは2000年2月に、0.5mg/ml未満の割合のトレンドは2002年5月に、それぞれ上昇から減少に転じる変曲点を示した。BACが0.5mg/ml以上の飲酒運転が占める割合のトレンドは、四輪運転者では1999年10月に、45歳未満運転者では2000年4月に、それぞれ上昇から減少に転じる変曲点を示した。二輪運転者では変曲点は無く、45歳以上運転者では2002年1月に増減無しから減少に転じる変曲点が示された。

**【考察】:**

1999年末から2000年初めにかけて見出されたトレンドの変曲点は、1999年11月に発生しメディアの注目を集めた事故が、世論を喚起し、飲酒運転行動の変化をもたらした可能性を示唆する。

## INTRODUCTION

Deterring drunk-driving is a challenging aspect of addressing the growing issue of road-traffic injuries worldwide, particularly in low- and middle-income countries that have an increasing number of motor vehicles and crashes.^1^^–^^3^ Some countries have considerably reduced the number of vehicle crashes resulting from drunk-driving.^1^^,^^2^ A study of the practices in such countries can help formulate effective measures in others.

Japan succeeded in reducing the number of fatal crashes involving drunk-driving, from 1276 in 2000 to 287 in 2010.^4^ This decline has been attributed mainly to legislative countermeasures.^5^^–^^7^ A road-traffic law amendment in June 2002 increased fines for drunk-driving by approximately 6-fold, lowered the punishable limit for blood alcohol concentration (BAC) from 0.5 mg/ml to 0.3 mg/ml, and increased the periods of license suspension and revocation. These measures were followed by an additional approximate doubling of fines in September 2007.

Although legal amendments introducing tougher penalties and lower BAC limits can reduce alcohol-related crashes if sanctions are imposed swiftly and with certainty,^8^^–^^11^ several time-series studies identified a decline in alcohol-related crashes even before such legislative initiatives, which suggests that changes in driver behavior preceded legislation.^6^^,^^12^^–^^14^ Introduction of severe penalties for drunk-driving often occurs after high-profile vehicle crashes caused by excessive drunk-driving. Such cases draw media attention, initiate social debate, and create momentum for legislation, which may spur pre-legislative changes in driver behavior.^13^^–^^16^

In Japan, 2 notable crashes that were caused by drunk-driving and resulted in the death of young children—1 in November 1999 and 1 in August 2006—raised public debate and media coverage, and prompted grass-roots initiatives for more-severe penalties for drunk-driving, which preceded amendments to the road-traffic law in 2002 and 2007 (Table 1). After both of these crashes, our previous time-series analysis identified an abrupt decline in fatal crashes involving drunk-driving.^6^ The change immediately after the 2006 crash was obvious, whereas the change after the 1999 crash was less pronounced.

**Table 1.** Timeline of key events

Time	Event
Nov. 1999	A high-profile crash in Tokyo: a heavily drunk truck driver caused a serious crash with a passenger car, and 2 young children died in the resulting fire
Apr. 2000	A high-profile crash in Kanagawa: 2 college students died after being hit by a car driven by a drunk, unlicensed driver
Nov. 2000	Petition signed by 160 000 people submitted to Minister of Justice
Oct. 2001	Petition signed by 370 000 people submitted to Minister of Justice
Jun. 2001	An amendment to the Road Traffic Law increasing penalties for drunk-driving passed the Diet
Nov. 2001	An amendment to the Criminal Law increasing the maximum penalty (from 5- to 15-year prison sentence) for driving resulting in death passed the Diet
Dec. 2001	An amendment to the Criminal Law increasing the maximum penalty (from 5- to 15-year prison sentence) for driving resulting in death came into effect
Jun. 2002	An amendment to the Road Traffic Law increasing penalties for drunk-driving went into effect
Aug. 2006	A high-profile crash in Fukuoka: a drunk driver hit a car with 3 young children and their parents; the car fell into water, and the children died
Sep. 2007	An amendment to the Road Traffic Law that further increased penalties for drunk-driving came into effect

In the time-series analyses used in the previous studies, events that were assumed to trigger trend changes (change-points) were selected from a group of events by the researchers in accordance with their hypothesis.^5^^,^^6^^,^^12^^–^^14^ Therefore, such analyses can test the selected hypotheses but cannot determine the most plausible one among several competing hypotheses. Our previous time-series analyses only tested the hypothesis that the 1999 crash was the onset of subsequent changes and ignored competing hypotheses that other notable events triggered the changes.^6^ Such events included the road-traffic law amendment introducing more severe penalties that passed the Diet (national legislature of Japan) in June 2001 and an amendment to the criminal law (introducing more-severe punishments for dangerous driving resulting in death) that came into effect in December 2001. In fact, the criminal law amendment was a potential rival cause for the change in drunk-driving behavior. Identifying the event that triggered the changes in driver behavior could provide practical information for other countries hoping to replicate such changes.

Therefore, the objective of the present study was to identify the event that was most likely to have triggered changes in trends of fatal crashes involving drunk-driving, with particular focus on the pre-legislative period. We used a joinpoint regression model, which identifies the occurrence of change-points that best fit the trend data, without a priori hypotheses. We also analyzed the trend according to driver age and vehicle type to ascertain whether these factors influenced drivers’ responses.

## METHODS

### Study settings

After a high-profile fatal crash in November 1999 and another in April 2000, public debate was aroused by media coverage of the bereaved families’ sorrow and the light sentences given to the offenders (Table 1).^17^ Subsequently, the bereaved families collected signatures in a petition calling for more severe penalties for such offenders. In total, 160 000 individuals signed the petition, which was submitted to the Minister of Justice in November 2000. By October 2001, 370 000 people had signed the petition.

Responding to these movements, the government amended the road-traffic law to increase the penalties for drunk-driving. The amendment passed the Diet in June 2001 and came into effect in June 2002. An amendment to the criminal law, which increased the maximum penalty for reckless driving resulting in death from 5 years’ to 15 years’ imprisonment, passed the Diet in November 2001 and came into effect in December 2001. This law applied only to car drivers.

### Design

To identify change-points in trends regarding crashes involving drunk-driving, we used joinpoint regression models to analyze nationally aggregated monthly data on fatal vehicle crashes in Japan from January 1995 to August 2006. The unit of analysis was fatal vehicle crashes, and the dependent variable of the regression models was the proportion of crashes involving drunk-driving.

### Data

From the Institute for Traffic Accident Research and Data Analysis, we obtained monthly police data relating to fatal crashes involving at least 1 vehicle (including 4-wheeled vehicles, motorcycles, and mopeds) and at least 1 death within 24 hours of the collision. The National Police Agency collected the data from all of Japan using a standardized format. In each crash, the police identified the driver primarily responsible for the crash (the “at-fault driver”) and recorded the driver's sex, age, and BAC level in addition to the vehicle type. The BAC was usually determined by breath-testing; alcohol consumption was determined by police investigation if breath-testing could not be carried out on-site owing to severe injury, escape, or refusal. The results of breath-testing were converted to BAC (0.25 mg/l exhaled is equivalent to a BAC of 0.5 mg/ml) and categorized as a BAC of 0.5 mg/ml or higher, a BAC greater than 0 but less than 0.5 mg/ml, BAC untested but alcohol consumption detected by police investigation (hereafter “untested”), no evidence of alcohol consumption (BAC = 0), or no information on alcohol consumption. The punishable BAC limit was 0.5 mg/ml (lowered to 0.3 mg/ml in June 2002), but driving with a BAC greater than 0.0 mg/ml but below the limits is also illegal and thus was recorded as drunk-driving.

We obtained monthly data on vehicle kilometers traveled (VKT)—which included information on passenger cars, buses, and trucks but did not include information on motorcycles—from the Ministry of Land, Infrastructure, Transport and Tourism. The data were used as the denominator of fatal crash rates per VKT to describe long-term trend of vehicle crashes; motorcycle crashes were included in the numerator, assuming the VKT of cars and motorcycles changed in parallel.

### Variables

The dependent variables of the joinpoint regression were the monthly proportions of fatal crashes involving drunk-driving by at-fault drivers (ie, the proportion of drunk-driving among at-fault drivers in fatal crashes), categorized by driver BAC levels. In accordance with previous studies we used proportions instead of number of crashes or rates, to control for extraneous factors affecting vehicle crashes and fatalities^6^^,^^18^^,^^19^; such factors include traffic volume (VKT), economic activities, gasoline prices, vehicle design, and emergency medical care. We assumed these factors were likely to evenly affect all fatal crashes (eg, increased traffic volume along with booming economies, indicating increased exposure to risk of crash, would similarly contribute to an increasing trend in both alcohol-related and non–alcohol-related fatal crashes). Crashes with no information regarding alcohol consumption of the at-fault driver were excluded from the denominator when calculating the proportions of drunk-driving by BAC level (BAC ≥ 0.5 mg/ml, 0 < BAC < 0.5 mg/ml, or untested) but were not excluded when calculating the proportion of those with no information.

### Statistical analysis

The analysis included data for the period from January 1995 to August 2006, just before the marked decrease in alcohol-related crashes identified in a previous study.^6^ This period did not include more recent years with relevant notable events because the main purpose was to identify pre-legislative trend change before June 2002, and later events would add trend segments without influencing the earlier trend. In addition, analysis over a longer period would demand that a joinpoint regression model encompass more change-points, which would require excessive computational time.

First, we used linear regression to adjust for the seasonal patterns of crashes involving drunk-driving. The dependent variable was the proportion of crashes involving drunk-driving; the independent variables were the months, which were entered into the regression model as dummy variables (with December as reference). Residuals were the variability that was not explained by seasonality. The residuals plus intercept provided the seasonally-adjusted proportions.

The joinpoint regression analyses were then performed, with the seasonally adjusted proportions as the dependent variables, and the time periods (ranging from t = 1 for January 1995 to t = 140 for August 2006) as the independent variables. We used a log-linear model, in which the dependent variable was log-transformed, assuming trends of geometric (equal ratio) rather than arithmetic (equal difference) sequences, as are usually seen in natural trends. Constant variance was assumed. The maximum number of change-points in trends (joinpoints) was set at 3. We assumed 2 notable events that triggered the trend changes during the study period: one was in the period before the road-traffic law amendment in June 2002 (main focus of this study) and the other was the law amendment. Our previous findings showed that the law amendment in June 2002 induced a pronounced baseline change (abrupt drop) in the trend rather than a simple slope change, which requires 2 change-points for model fitting.

The joinpoint regression model is a combination of several trend segments, in which adjacent trends meet in a joinpoint, and the number and place of the joinpoints are determined without preselected hypothesized change-points. Joinpoint regression analyses initially test the null model with no joinpoint against alternative models and determine whether and where more joinpoints should be added, using a permutation test to best fit the model to the data.^20^ We used SPSS version 18 (SPSS Inc., Chicago, IL, USA) for the linear regression analyses and the Joinpoint Regression Program 3.4.3 (National Cancer Institute) for the joinpoint regression analyses.

We analyzed the trends in the proportions of drivers with different BAC levels (BAC ≥ 0.5 mg/ml, 0 < BAC < 0.5 mg/ml, or untested), and of those with no information. Then we conducted stratified analyses by vehicle type (car or motorcycle) of the at-fault driver and by driver age group (<45 or ≥45 years) only for the proportions of drivers with a BAC of 0.5 mg/ml or higher, which showed a significant pre-legislative trend change in the whole-group analyses. The small number of female drivers with a BAC of 0.5 mg/ml or higher made it impossible to perform stratification by sex (Table 2). The small number of fatal crashes in each month did not allow us to have more than 2 categories for each stratification. The car category included all 4-wheel vehicles, and the motorcycle category covered all 2-wheel motor vehicles including mopeds. The age categories were collapsed into 2 based on trend similarities in more-detailed age categories.

**Table 2.** Number of crashes involving drunk-driving from 1995 to 2006

	BAC ≥ 0.5 mg/ml		BAC < 0.5 mg/ml		Untested but detected		No evidence of alcohol use		No information on alcohol use^a^	
				
	*n*	%	*n*	%	*n*	%	*n*	%	*n*	%
**Year**										
1995	917	10.2	251	2.8	223	2.5	7607	84.5	229	2.5
1996	862	10.3	205	2.4	229	2.7	7083	84.5	232	2.7
1997	826	10.1	218	2.7	196	2.4	6954	84.9	226	2.7
1998	875	11.2	192	2.5	200	2.6	6558	83.8	277	3.4
1999	883	11.4	217	2.8	157	2.0	6502	83.8	201	2.5
2000	872	11.1	238	3.0	166	2.1	6554	83.7	194	2.4
2001	743	9.9	249	3.3	199	2.7	6305	84.1	218	2.8
2002	621	8.7	211	3.0	165	2.3	6127	86.0	200	2.7
2003	483	7.2	153	2.3	144	2.2	5897	88.3	162	2.4
2004	463	7.2	138	2.2	109	1.7	5694	88.9	99	1.5
2005	447	7.5	153	2.6	107	1.8	5285	88.2	118	1.9
2006^b^	303	8.6	111	3.1	61	1.7	3061	86.6	54	1.5
**Sex**										
Male	7709	10.5	2173	3.0	1800	2.4	61 797	84.1	2019	2.7
Female	586	4.6	163	1.3	156	1.2	11 830	92.9	191	1.5
**Age**										
<25	2106	9.7	779	3.6	620	2.9	18 165	83.8	679	3.0
25–44	3709	12.3	1014	3.4	835	2.8	24 692	81.6	784	2.5
45–64	2042	8.8	441	1.9	430	1.9	20 185	87.4	533	2.3
≥65	438	3.9	102	0.9	71	0.6	10 585	94.5	214	1.9
**Vehicle**										
Car	7437	10.0	2176	2.9	1586	2.1	63 199	84.9	1759	2.3
Motorcycle	858	7.3	160	1.4	370	3.1	10 428	88.3	451	3.7

^a^Those with no information on alcohol consumption were excluded from the denominator in calculating the proportions of the other categories; calculation of the proportion of those with no information used the total number of crashes as the denominator.

^b^The data for 2006 include crashes only between January and August.

## RESULTS

**Figure 1.**
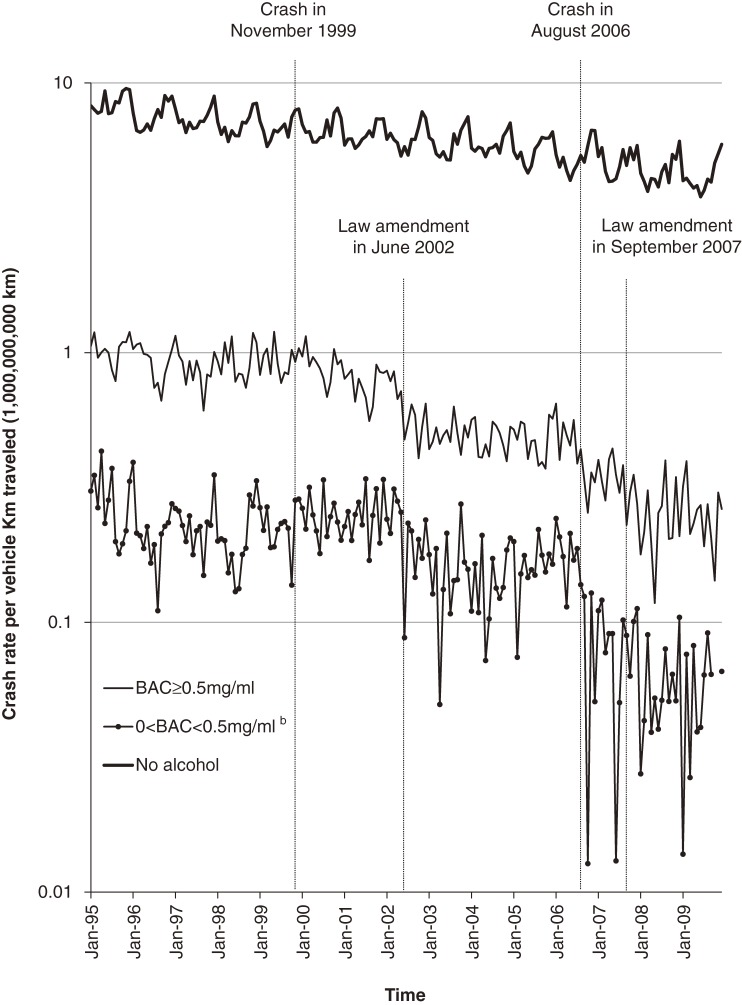
Fatal crash rate^a^ per vehicle kilometer traveled, 1995–2009. ^a^Crude rates without seasonal adjustment are shown. The numerators included the number of crashes by motorcycles, although the denominators did not include the kilometers traveled by motorcycle because such data were unavailable. ^b^The rate of crashes involving drunk-driving with blood alcohol concentration (BAC) <0.5 mg/ml was 0 in October and November in 2009.

Figure 1 shows fatal crash rates per VKT by BAC level. Fatal crashes that did not involve drunk-driving showed a consistent declining trend; crashes involving drunk-driving showed steeper declines, particularly after the law amendment in 2002 and the 2006 crash. During the study period, the number of fatal crashes decreased markedly (Table 2), from 9227 in 1995 to 6110 in 2005. Among at-fault drivers, males and car drivers accounted for the great majority—85.4% and 86.1%, respectively. Drivers aged 65 years or older accounted for a small fraction (12.9%). Male versus female drivers, drivers younger than 65 years versus those 65 years or older, and car drivers versus motorcyclists were more likely to have a positive BAC.

**Figure 2.**
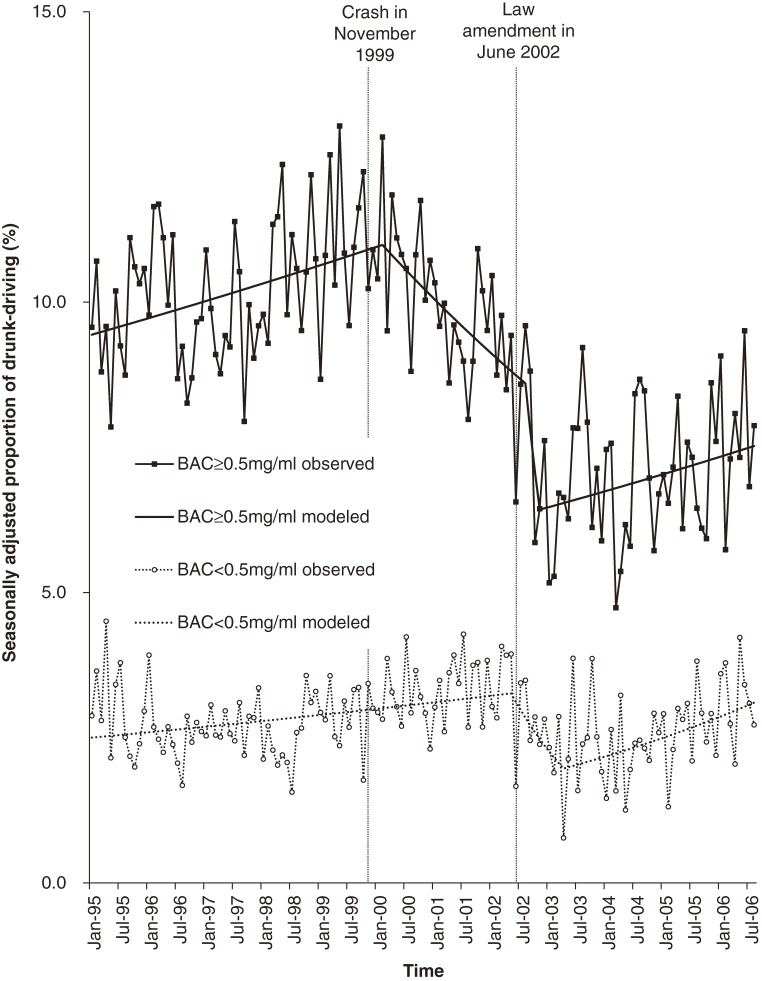
Proportion of drunk-driving by BAC level among at-fault drivers in fatal crashes from January 1995 to August 2006.

Figure 2 shows the proportions of drunk-driving by BAC level among all at-fault drivers. The proportion of those with a BAC of 0.5 mg/ml or higher showed an increasing trend of 0.25% per month (95% CI, 0.07% to 0.43%; hereafter the trend indicates a month-over-month percentage change rather than a percentage point difference) until the change-point of February 2000 (95% CI, March 1998 to June 2001), turning to a declining trend of −0.81% (95% CI, −1.36 to −0.26), then to a steeper declining trend of −9.25% (95% CI, −36.5 to 29.3) in August 2002 (95% CI, June 2001 to June 2004), and subsequently to an increasing trend of 0.35% (95% CI, 0.06 to 0.64) in November 2002 (95% CI, August 2002 to September 2004). The proportion of those with a BAC greater than 0.0 but less than 0.5 mg/ml showed an increasing trend of 0.30% per month (95% CI, 0.11 to 0.49) until the change-point of May 2002 (95% CI, April 2001 to February 2003), turning to a declining trend of −4.49% (95% CI, −9.15 to 0.41), then to an increasing trend of 1.14% (95% CI, 0.52 to 1.77) in April 2003 (95% CI, September 2002 to September 2004). The proportion of those untested showed a decreasing trend of −0.34% per month (95% CI, −0.46 to −0.23) with no change-point, and the proportion of those with no information on alcohol consumption showed no significant trend until a change-point of April 2002 (95% CI, May 1998 to May 2003), turning to a decreasing trend of −1.41% (95% CI, −2.00 to −0.81) (data not shown in figure).

**Figure 3.**
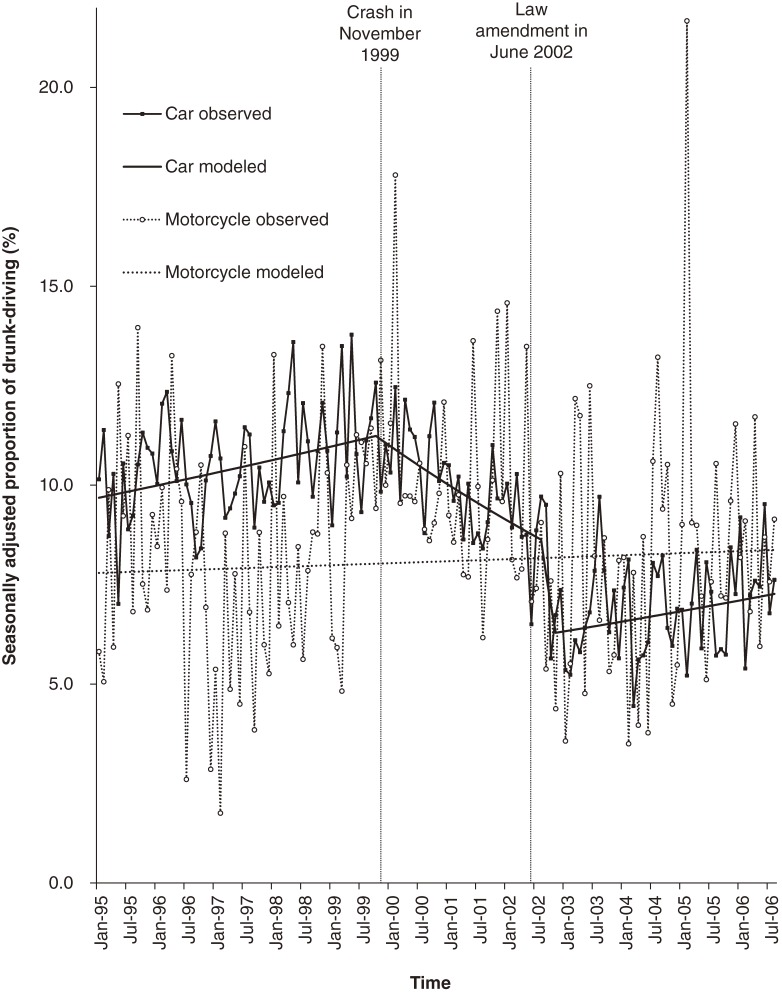
Proportion of drivers with a BAC of 0.5 mg/ml or higher among at-fault drivers in fatal crashes by vehicle type from January 1995 to August 2006.

Figure 3 shows the proportion of drunk-driving with a BAC of 0.5 mg/ml or higher among at-fault drivers by vehicle type. The proportion among car drivers showed an increasing trend of 0.26% per month (95% CI, 0.04 to 0.48) until the change-point of October 1999 (95% CI, November 1997 to May 2001), turning to a decreasing trend of −0.77% (95% CI, −1.27 to −0.28), then to a steeper declining trend of −10.0% (95% CI, −38.8 to 32.4) in August 2002 (95% CI, May 2001 to June 2003), and subsequently to an increasing trend of 0.32% (95% CI, 0.01 to 0.64) in November 2002 (95% CI, August 2002 to March 2006). The proportion of drunk-driving among motorcyclists showed neither a significant trend nor a change-point.

**Figure 4.**
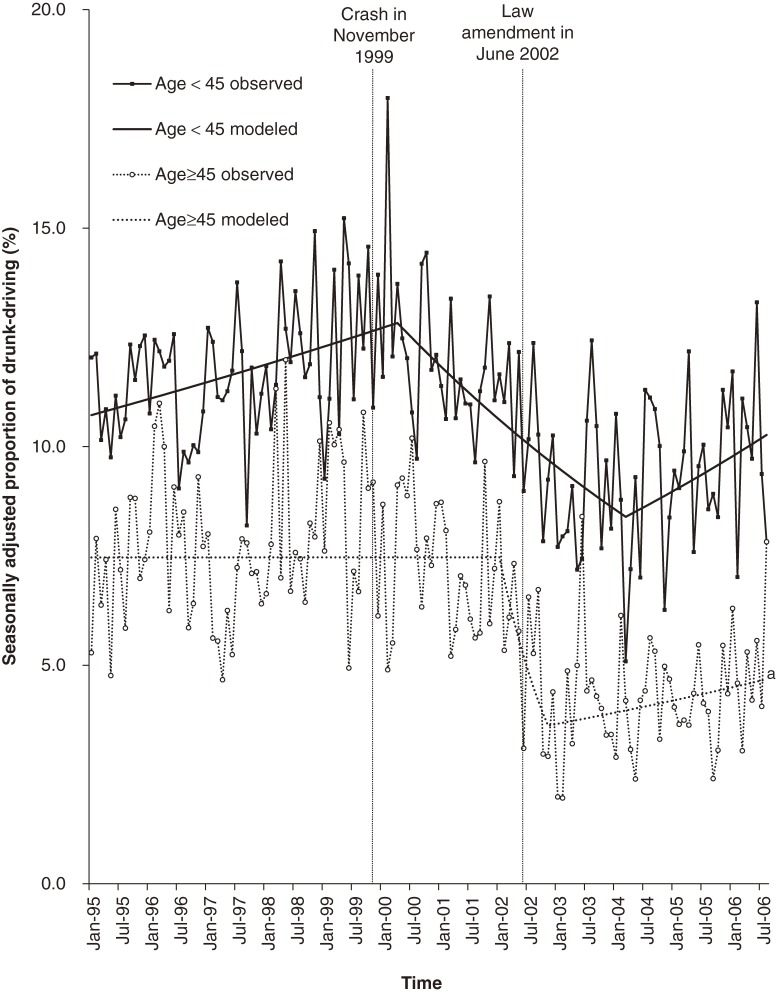
Proportion of drivers with a BAC of 0.5 mg/ml or higher among at-fault drivers in fatal crashes by age group from January 1995 to August 2006.

Figure 4 shows the proportion of drunk-driving with a BAC of 0.5 mg/ml or higher among at-fault drivers by age group. The proportion among drivers younger than 45 years showed an increasing trend of 0.29% per month (95% CI, 0.09 to 0.48) until the change-point of April 2000 (95% CI, March 1999 to June 2002), turning to a decreasing trend of −0.90% (95% CI, −1.21 to −0.58), then to an increasing trend of 0.70% (95% CI, 0.06 to 1.33) in March 2004 (95% CI, August 2002 to December 2004). The proportion among drivers 45 years or older showed no significant trend until the change-point of January 2002 (95% CI, April 2000 to September 2002), turning to a decreasing trend of −6.99% per month (95% CI, −12.9 to −0.70), then to a non-significant trend in November 2002 (95% CI, May 2002 to March 2006).

## DISCUSSION

The proportion of fatal crashes involving drunk-driving with a BAC of 0.5 mg/ml or higher by at-fault drivers showed a change-point around the turn of the year from 1999 to 2000 among all drivers, car drivers, and drivers younger than 45 years. In contrast, the proportion of drunk-driving with a BAC greater than 0 but less than 0.5 mg/ml among all drivers and the proportion of those with a BAC of 0.5 mg/ml or higher among drivers 45 years or older showed a change-point around the time of the road-traffic law amendment in June 2002; those with a BAC of 0.5 mg/ml or higher among motorcycle drivers did not show any change-point.

The findings are consistent with the hypothesis that the declining trend in alcohol-related crashes started with the news coverage of the high-profile fatal crash in November 1999 and possibly an April 2000 crash. The December 2001 amendment to the criminal law, a potential change-point, was unlikely to be at the onset of the declining trend. Severe penalties based on the amended criminal law applied only to drivers who had caused a fatal crash, which were extremely rare cases. Therefore, drivers might not have had a perception that there was an increased likelihood of being arrested and punished, which is necessary for severe sanctions to be effective.^8^

We do not claim that news coverage alone was able to reduce alcohol-related crashes. Rather, the media might have initiated a series of movements by helping to shape public opinion by arousing public anger against drunk-driving and converting it into support for policy action.^15^^,^^21^^–^^24^ The observed long-term declining trend is attributable to several events, including repeated legislative interventions. The increasing trend after the law amendment in 2002 suggests that the effects of the intervention waned. The additional events (the 2006 crash and the intervention in 2007) might have contributed to a further decline, though the present study did not assess these events. In addition, without a certain level of social concern or public support, legislation with severe sanctions could not have been so readily accepted and thus might not have achieved long-term success.^12^^,^^14^^,^^25^

The present study identified a clear pre-legislative trend change only in the proportion of drunk-driving with a BAC of 0.5 mg/ml or higher, particularly among car drivers and those younger than 45 years. The pre-legislative responsive behavioral changes not mandated by the law may have been partial (avoiding heavy drunk-driving), occurring among individuals who were sensitive to public debate and societal concerns, such as younger car drivers. The lack of a response among motorcyclists may have resulted from the fact that the debate focused on car drivers, since drunk car drivers caused the high-profile crashes. In contrast, the coercive power of the legal amendment in June 2002 might have changed behavior among less sensitive people (older adults) and, in combination with the lowered punishable BAC limit, reduced drunk-driving at lower alcohol consumption levels.

Evidence supports the notion that older adults with an established lifestyle and behavior are less responsive than younger people to societal change. A time-series analysis in the United States showed a greater decline in drunk-driving among young drivers (<35 years) than among older drivers in response to an increased media campaign.^15^ Other factors that may have contributed to low responsiveness among older adults include the higher prevalence of problem drinkers among males in their 40s and 50s in Japan^26^ and the increased confidence in one’s driving ability with age, even after consuming alcohol.^27^

Although our analyses did not directly control for the various factors that affect vehicle crashes and fatalities, such factors are unlikely to explain the present findings. Our model used the proportion of drunk-driving among at-fault drivers in fatal crashes as the dependent variable to control for factors such as traffic volume, economic activities, gasoline prices, vehicle design, and medical care. This method was based on an assumption that these factors similarly affect both alcohol-related and non–alcohol-related crashes. Contrary to this assumption, some of these factors, and other factors such as overall alcohol consumption, may disproportionately affect alcohol-related crashes. They could not, however, explain the abrupt change found in this study, given the gradual changes in these factors during the study period. Alcohol consumption in Japan increased in the 1980s, plateaued during the 1990s, and started to decrease in 2002.^28^ This change in alcohol consumption may partly explain the long-term declining trends in drunk-driving crashes after 2002.

The models did not consider police-enforcement activities. However, the police usually undertake only short-term (typically 1-week) crackdown campaigns after high-profile crashes.^6^ The number of drivers charged with drunk-driving, which had been quite constant for more than 10 years, decreased by 24% in 2000 (ie, before the law amendment)^29^^–^^31^; this suggests that the reduction in drunk-driving occurred without an increased likelihood of being caught. Although some drivers may have equated law enactment with automatic enforcement and changed their behavior when the law amendments were reported by the media, the observed change started many months before the amendments passed the Diet (road-traffic law in June 2001 and criminal law in November 2001).

Some crash cases lacked BAC-level data from at-fault drivers (untested or no information category). If those with a high BAC increasingly fell into these categories, this might have produced an apparent decrease in the proportion of those with BAC of 0.5 higher or a BAC greater than 0 but less than 0.5 mg/ml. This is unlikely, however. The proportions of those untested or with no information did not show any increasing change in the present study. Our previous time-series analysis showed an increasing change in the trend of untested cases after the November 1999 crash; however, the increase was far less than the decrease in those with a BAC of 0.5 mg/ml or higher.^6^

The present study did not quantitatively or qualitatively examine changes in the news coverage of alcohol-related crashes or attempt to determine how such changes influenced the trend in the crashes. Therefore, further research using statistical and content analyses is needed to examine changes in news coverage. If changes are detected, they should be linked with the trend change identified in the present study.^15^^,^^22^

In conclusion, the present study identified the onset of a declining trend in fatal crashes involving drunk-driving, namely, around the same time as some high-profile crashes. Although further research should address how the media influenced driver behavior, our findings suggest that the high-profile crashes helped spark public debate, which prompted movements that resulted in policy action, such as amendment of the road-traffic law, and induced pre-legislative behavioral change among drivers. Therefore, it is important to describe and examine the role the media played in stimulating the sequence of changes.

## ONLINE ONLY MATERIALS

Abstract in Japanese.
