# Supplementary material for: Onset of a Declining Trend in Fatal Motor Vehicle Crashes Involving Drunk-driving in Japan
Source: J Epidemiol. 2013 May 5;23(3):195–204. doi: 10.2188/jea.JE20120134 (PMC3700255; doi:10.2188/jea.JE20120134)
Supplement: Abstract in Japanese. [file je-23-195-s001.pdf]

わが国の飲酒運転による死亡事故が減少傾向に転じた時期についての検討

中原慎二[1]、片野田耕太[2]、市川政雄[3]

1 聖マリアンナ医科大学
2 国立がん研究センター　がん対策研究センター
3 筑波大学　医学医療系

【背景】日本におけるアルコールが関与する死亡交通事故は、2002 年 6 月の道路交通法改正による飲酒運転に対する罰則の強化と、罰則適用基準値の引き下げにより、大きく減少した。しかし、先行研究ではこの減少傾向が法改正よりも前に始まった可能性が指摘されている。この減少傾向の原因については十分な分析が行われていない。

【方法】分析には 1995 年 1 月から 2006 年 8 月までの、警察庁集計による月別死亡交通事故データを用いた。死亡事故の第一当事者（当該事故で最も責任の重いと考えられた運転者）が飲酒をしていた割合を従属変数とし、そのトレンドの変曲点をジョインポイント回帰分析により求めた。分析はまず、全第一当事者に占める血中アルコール濃度（Blood alcohol concentration: BAC）が 0.5mg/ml 以上と 0.5mg/ml 未満（道路交通法は血中濃度にかかわらず酒気を帯びての運転を禁じており、罰則適用基準値未満でも飲酒運転として事故データに記録される）の割合について行った。次に、車種別（四輪車、二輪車）、年齢別（45 歳以上、45 歳未満）に層別化し、0.5mg/ml 以上の割合について分析した。

【結果】全第一当事者に占める、BAC が 0.5mg/ml 以上の飲酒運転の割合のトレンドは 2000 年 2 月に、0.5mg/ml 未満の割合のトレンドは 2002 年 5 月に、それぞれ上昇から減少に転じる変曲点を示した。BAC が 0.5mg/ml 以上の飲酒運転が占める割合のトレンドは、四輪運転者では 1999 年 10 月に、45 歳未満運転者では 2000 年 4 月に、それぞれ上昇から減少に転じる変曲点を示した。二輪運転者では変曲点は無く、45 歳以上運転者では 2002 年 1 月に増減無しから減少に転じる変曲点が示された。

【考察】1999 年末から 2000 年初めにかけて見出されたトレンドの変曲点は、1999 年 11 月に発生しメディアの注目を集めた事故が、世論を喚起し、飲酒運転行動の変化をもたらした可能性を示唆する。

キーワード：飲酒、日本、メディア、交通外傷、回帰分析
